# Web-Based Cognitive Behavior Therapy for Depression With and Without Telephone Tracking in a National Helpline: Secondary Outcomes From a Randomized Controlled Trial

**DOI:** 10.2196/jmir.1859

**Published:** 2012-06-27

**Authors:** Louise Farrer, Helen Christensen, Kathleen M Griffiths, Andrew Mackinnon

**Affiliations:** ^1^Centre for Mental Health ResearchThe Australian National UniversityCanberra, ACTAustralia; ^2^Biostatistics UnitOrygen Youth Health Research CentreUniversity of MelbourneMelbourne, VICAustralia

**Keywords:** eHealth, CBT, cognitive behavior therapy, depression, telephone support

## Abstract

**Background:**

An earlier report indicated that callers to a telephone counseling service benefited from the addition of an evidence-based Web intervention for depression. It is not known whether the Web intervention would also lower alcohol use and stigma, or improve quality of life and knowledge of depression and its treatments.

**Objective:**

To report the secondary outcomes of a trial of a Web-based cognitive behavior therapy (CBT) intervention for depression, including hazardous alcohol use, quality of life, stigma, depression literacy, and CBT literacy.

**Methods:**

We recruited a sample of 155 callers to Lifeline, a national telephone counseling service in Australia, who met the criteria for moderate to high psychological distress. Participants were randomly assigned to 1 of 4 conditions: (1) Web CBT plus weekly telephone tracking, (2) Web CBT only, (3) weekly telephone tracking only, and (4) neither Web CBT nor telephone tracking. Participants were assessed at preintervention, postintervention, and 6 and 12 months postintervention.

**Results:**

At postintervention, participants who completed the Web intervention either with or without telephone support had lower levels of hazardous alcohol use (without tracking: *P *= .008, effect size = 0.23; with tracking: *P *= .003, effect size = 0.26), improved quality of life (without tracking: *P *= .001, effect size = 0.81; with tracking: *P *= .009, effect size = 0.63), and improved CBT literacy (without tracking: *P *= .01, effect size = 0.71; with tracking: *P *< .001, effect size = 0.80) compared with those who did not receive the Web intervention or telephone support. Results for quality of life and CBT literacy were maintained at 6- and 12-month’s follow-up, but differences in hazardous alcohol use were not significantly different between conditions at 6 and 12 months. Although omnibus tests for depression literacy and stigma were nonsignificant, contrasts revealed that those in the Web-only condition showed significantly lower levels of stigma than participants in the control condition at postintervention. This was true for participants in the Web-only and Web plus tracking conditions at 6 months. Similarly, those in the Web-only and Web plus tracking conditions had significantly higher depression literacy at postintervention, and this was maintained in the Web-only condition at 6-months’ follow-up. No significant differences were found in depression literacy and stigma between conditions at 12 months.

**Conclusions:**

Evidence-based Web interventions for depression can be effective not only in reducing depression symptoms but also in improving other health outcomes, including quality of life, hazardous alcohol use, and knowledge about effective strategies for depression self-management.

**Trial Registration:**

International Standard Randomized Controlled Trial Number (ISRCTN): 93903959; http://www.controlled-trials.com/ISRCTN93903959/ (Archived by WebCite at http://www.webcitation.org/65y61nSsH)

## Introduction

Web-based, self-administered cognitive behavior therapy (CBT) programs have been shown to be effective in reducing symptoms of depression [1-4]. Reviews have suggested that the effects of these programs may be enhanced by the provision of guidance from therapists [4]. Based on their recent review of the evidence, Newman and colleagues assert that Web interventions with ongoing assistance from a therapist are superior to other modes of online delivery in the treatment of clinical levels of depression [5]. They argue that Web interventions with less-intensive support (eg, those that involve either no human support or periodic monitoring from a nonspecialist) are more appropriate in the treatment of people with subthreshold mood disorders. However, Titov and colleagues demonstrated that technician (nonspecialist) -administered guidance was as effective as therapist-administered guidance in a Web-based treatment for participants with major depressive disorder [6]. Recently, in a randomized controlled trial of a Web-based CBT program for depression, we found significant reductions in depression symptoms and caseness, both when guidance was provided by a trained volunteer and when no guidance was provided at all, with no significant difference in the effect of the two [7].

While there is evidence for the efficacy of these programs for depression symptoms, less is known about the effects of Web-based programs, guided or not, on secondary outcomes such as alcohol use, quality of life, stigma about depression, or knowledge of depression and its treatment. Accordingly, the current study sought to extend the previously reported outcomes by Farrer and colleagues [7] by examining the effects of the intervention on the following outcomes: hazardous alcohol use, quality of life, stigma, depression literacy, and CBT literacy.

These outcomes are important for several reasons. Depression and hazardous alcohol use frequently co-occur in the general population and in clinical samples [8]. The prevalence of lifetime comorbidity of major depression and alcohol use disorders has been estimated at between 13% and 18% [9,10]. Evidence suggests that depression can increase the likelihood of heavy alcohol use, particularly in women [11]. In recognition of the common causal or etiological mechanisms that may underpin both depressive and alcohol use disorders, CBT is the predominant psychological treatment approach for these conditions. Moreover, computer-based CBT-based interventions have been shown to be effective in the treatment of those with comorbid depression and alcohol use disorders [12].

 Quality of life has an evident negative association with depression. Individuals with depression have been shown to have lower levels of functioning and well-being than those with other chronic conditions [13]. Depression is also associated with impaired social and occupational roles [14,15]. Thus, quality of life is an important metric for assessing the impact of an intervention beyond the alleviation of symptoms. It has been argued that a comprehensive assessment of the effectiveness of an intervention should include both the degree to which the intervention prevents or treats symptoms and the degree to which it enhances the basic elements of well-being [16].

In addition to cognitive restructuring and behavioral activation techniques, comprehensive CBT interventions for depression include a psychoeducational component designed to improve clients’ knowledge and understanding of their illness. Education about the signs of depression may improve an individual’s ability to detect and respond appropriately to symptoms within themselves and others. There is also evidence that *passive *psychoeducation can have a positive effect on depression symptoms (see [17]). Depression literacy is a term used specifically to describe an individual’s awareness of the causes, epidemiology, symptoms, diagnosis, and treatment of depression. Therefore, level of depression literacy is a useful metric for assessing the impact of the psychoeductional component of a CBT-based intervention.

 Many individuals with depression may not seek treatment or drop out of treatment prematurely due to the stigma commonly associated with mental health disorders [18]. To reduce the prevalence of depression stigma, educational interventions have been developed to target the common misconceptions held by members of the general public as well as specific groups such as health care professionals [19]. Moreover, individuals with mental disorders have themselves been shown to hold negative views about mental illness [20]. Therefore, educational interventions may effectively reduce stigmatizing attitudes held by those who experience depression. In a previous trial, Web-based psychoeducation was shown to effectively reduce stigma in a community sample with more intense depression symptoms [21], but the effects in a sample with higher needs has not been investigated.

Having established that the Web-based intervention in our recent trial was effective for depression, we hypothesized in this study that Web-based psychoeducation and CBT would be more effective than the control condition in (1) reducing hazardous levels of alcohol use and (2) improving quality of life. Given the psychoeducational and therapeutic content of the intervention, we also expected that the intervention would be effective in (3) reducing stigma relating to depression, (4) improving depression literacy, and (5) improving CBT literacy. We hypothesized that the combination of tracking with the Web intervention would be superior to the other 3 conditions, given the weight of evidence from previous research.

## Methods

Data for this study were collected as part of a larger randomized controlled trial of the impact of a Web-based CBT and psychoeducation intervention on depression symptoms [7]. For a full description of the design and methods of the trial, see Farrer et al [7].

### Participants

Participants were 155 callers to Lifeline, Australia’s 24-hour telephone counseling service, recruited from counseling centers in four major Australian cities between July 2007 and January 2009. A total of 910 callers agreed to be screened by telephone. Of these, 142 (15.6%) were subsequently unable to be contacted, 61 (7%) were later unwilling to participate, and 337 (37%) did not meet eligibility criteria. Respondents were not eligible for inclusion in the trial if they (1) scored less than 22 on the Kessler Psychological Distress Scale [22] (138/337, 41%), (2) had a self-reported diagnosis of schizophrenia or bipolar disorder (89, 26%), or (3) did not have Internet access (67, 20.0%). Ineligible participants were offered brochures sent by mail containing information about the Web intervention used in the trial. Of the 370 people eligible for inclusion in the trial, 155 completed informed consent procedures and preintervention assessments, and were randomly assigned to the trial conditions.

### Procedure

Following screening and informed consent procedures, preintervention data were obtained through a self-report questionnaire mailed to participants. We used a block randomization procedure with stratification based on site of recruitment and severity of psychological distress at screening. Allocation of participants to trial conditions was conducted independently by a research assistant not involved in the day-to-day running of the trial. Following randomization, all participants were contacted by telephone and were mailed the relevant materials for their allocated condition.

### Intervention and Trial Conditions

The Web-only intervention consisted of Web-based psychoeducation (in week 1 provided by BluePages: bluepages.anu.edu.au) combined with Web-based CBT (in weeks 2–6 provided by MoodGYM: moodgym.anu.edu.au). Both of these Web programs have been shown to reduce depression symptoms in community users [23]. A printed manual containing week-by-week instructions for accessing the Web programs (via a login) was mailed to participants at the start of the trial.

In the Web with tracking condition, participants completed the Web intervention and also received a weekly 10-minute telephone call from a telephone counselor. The purpose of these calls was to address any issues associated with the participants’ use of the intervention.

In the tracking-only condition, participants received a weekly 10-minute telephone call from a telephone counselor. These calls focused on various environmental and lifestyle factors associated with depression.

In the control condition, participants received neither the tracking nor Web interventions. Participants in this condition were wait-listed to receive the Web-only intervention following completion of the 6-month follow-up.

Participants in all 4 conditions were free to use the Lifeline telephone counseling service as needed, which provided usual emergency or support services. Any use of this service during the intervention period was additional to the 10-minute telephone calls offered as part of the Web with tracking and tracking-only intervention conditions. The 10-minute intervention calls were scripted and not intended to provide any form of counseling.

### Measures

Data were obtained by self-report questionnaires mailed to participants at preintervention, postintervention (6 weeks following the preintervention assessment), 6 months postintervention, and 12 months postintervention.

#### Hazardous Alcohol Use

We measured hazardous alcohol use using a 5-item version of the Alcohol Use Disorders Identification Test (AUDIT) [24]. Item scores were summed to provide a total scale score ranging from 0 to 20, with higher scores indicating greater hazardous alcohol use. A score of 5 or above indicates hazardous alcohol consumption [25]. Hazardous alcohol consumption is defined as a pattern of drinking behavior that increases the risk of harmful consequences to the drinker or others [26]. Hazardous drinking behavior does not necessarily reflect the presence of an alcohol use or dependence disorder, although it is a strong risk factor [24]. The internal consistency of the 5-item AUDIT in the current sample was 0.87 (n = 107).

#### Quality of Life

We assessed quality of life using the EUROHIS-QOL 8-item index [27]. The EUROHIS-QOL is composed of 8 items designed to measure the psychological, physical, social, and environmental aspects of quality of life. Items for the EUROHIS-QOL were extracted from the larger World Health Organization Quality of Life (WHOQOL) -100 and WHOQOL-BREF scales, both of which have well-established psychometric properties [28,29]. Item responses were summed, producing a total score ranging from 0 to 32, with higher scores indicating higher quality of life. The internal consistency of the EUROHIS-QOL for the current sample was 0.79 (n = 151).

#### Stigma

We assessed stigma using the personal stigma subscale of the Depression Stigma Scale [21,30]. The personal stigma subscale of the Depression Stigma Scale is composed of 9 items reflecting the participants’ personal attitudes toward people with depression. The items were scored on a 5-point scale ranging from strongly agree to strongly disagree, yielding a summed total scale score ranging from 0 to 36, with higher scores indicating higher stigma. In the current sample, the internal consistency of this scale was 0.74 (n = 151).

#### Depression Literacy

We assessed depression literacy using 11 items from a 22-item scale that we had developed for use in a previous community-based Web intervention trial [21]. Participants were provided with 11 statements relating to depression and asked to rate them as either true or false. Example statements are “Reckless and foolhardy behavior is a common sign of depression” (false) and “Antidepressants are addictive” (false). Correct responses received a score of 1, and scores were summed to provide a total depression literacy score ranging from 0 to 11, with higher scores indicating higher depression literacy. The internal consistency of this scale for the current sample was 0.69 (n = 151). This scale was previously shown to be sensitive to the effects of an online depression psychoeducation intervention [21,31].

#### Cognitive Behavior Therapy Literacy

Knowledge of the principles of CBT was assessed using 8 items from an 18-item scale that we had developed for use in a previous community-based Web intervention trial [23]. The 8 items were statements about CBT that participants were asked to rate as either true or false. Example statements are “I should automatically believe my thoughts because they will more often than not be accurate” (false) and “The statement ‘I’m a stupid idiot’ is an example of labeling” (true). Correct responses received a score of 1, and scores from the 8 items were summed to provide a total CBT literacy score ranging from 0 to 8, with higher scores indicating higher CBT literacy. The internal consistency of this scale for the current sample was 0.69 (n = 148). This scale was previously shown to be sensitive to the effects of an online CBT intervention [23].

### Statistical Analysis

We analyzed data using SPSS release 18.0.1 for Windows (IBM Corporation, Somers, NY, USA) and Stata 10.1 (StataCorp LP, College Station, TX, USA). A significance level of .05 was used for all outcome variables. We analyzed all outcomes on an intention-to-treat basis. With the exception of alcohol use, all variables had distributions suitable for standard statistical methods. The stigma, quality of life, depression literacy, and CBT literacy variables were analyzed using linear mixed-models repeated-measures analysis of variance, with measurement occasion as a within-groups factor and intervention condition as a between-groups factor. We modeled within-person variation using an unstructured covariance matrix. Mixed modeling allows the use of all available data for each participant (as opposed to substituting missing data with estimated values) [32]. Imputing missing values is potentially problematic, as it can increase the risk of type I error by reducing error variance and artificially increasing degrees of freedom.

The AUDIT had an exponential-shaped distribution, with most participants scoring zero and diminishing numbers with higher scores. Dichotomization at the cut-off for hazardous drinking was not feasible, as it resulted in cells with no variance, having all observations below the cut-off, and low statistical power. Accordingly, we analyzed AUDIT scores using random-intercept Poisson regression. Poisson regression model counts, in this case, were the total numbers of thresholds (divisions between response categories) crossed on the AUDIT items [33]. The model included intervention and occasion as predictors and an intercept for each participant. The latter random parameters account for within-person correlation of responses over occasions and for overdispersion. Overdispersion refers to observations having greater variance than the mean, as implied by the Poisson model. This analysis was undertaken using the gllamm (generalized linear latent and mixed models) procedure in Stata [34].

For each outcome, we used planned contrasts to compare intervention and control groups postintervention, and at 6- and 12-months’ follow-up. We estimated effect sizes by dividing the mean difference between conditions postintervention and at 6- and 12-months’ follow-up by the pooled standard deviation of the groups. Data inspection prior to analysis revealed that two participants in the Web with tracking condition had aberrant patterns of change, each having moderate scores on the Center for Epidemiologic Studies Depression Scale at preintervention and extremely severe scores at postintervention. In one case, we were able to link this to the diagnosis of a life-threatening illness. Due to distortion of the variance and violation of the assumption of normality [35], these participants were removed for the main data analysis. The achieved sample size of 155 participants maintained power above 80% to detect differences between treatment arms of 0.5 SD.

## Results

### Participation Rates

Of 370 eligible respondents, 155 completed informed consent procedures and preintervention assessments, and were randomly assigned to trial conditions, resulting in a 41.9% acceptance rate. Of the 155 participants, 107 (69.0%) returned postintervention surveys, and 92 (59%) completed the 6-month follow-up. Table 1 shows observed means for outcomes across conditions at preintervention, postintervention, 6-month follow-up, and 12-month follow-up. At preintervention, we found no significant differences between participants in each condition on any of the variables of interest: quality of life (*F*_3,151 _= .10, *P *= .96), hazardous alcohol use (*F*_3,103 _= .55, *P *= .65), stigma (*F*_3,147 _= 1.21, *P *= .31), depression literacy (*F*_3,147 _= .98, *P *= .40), and CBT literacy (*F*_3,144_ = 1.41, *P * .24).

**Table 1:** Observed mean scores for hazardous alcohol use, quality of life, stigma, depression literacy, and cognitive behavioral therapy (CBT) literacy for each intervention condition across measurement occasions.

Measure	Condition	Measurement occasion							
		Preintervention		Postintervention		6-month follow-up		12-month follow-up	
		n^a^	Mean (SD)	n^a^	Mean (SD)	n^a^	Mean (SD)	n^a^	Mean (SD)
**Hazardous alcohol use**									
	Web only	38	4.53 (5.37)	27	2.78 (4.12)	23	3.22 (4.47)	21	3.57 (5.20)
	Web with tracking	45	4.73 (5.76)	17	2.35 (3.10)	20	2.85 (2.70)	14	2.36 (2.37)
	Tracking only	36	3.81 (4.57)	31	3.90 (4.31)	24	3.21 (4.71)	22	3.82 (4.90)
	Control	34	3.09 (4.27)	24	3.92 (5.63)	21	2.57 (3.96)		—
** Quality of life **									
	Web only	38	12.24 (5.72)	27	16.70 (6.83)	23	17.30 (7.38)	21	17.90 (5.22)
	Web with tracking	45	12.42 (5.22)	17	14.71 (6.75)	20	16.10 (6.27)	14	15.86 (6.86)
	Tracking only	37	12.73 (6.64)	33	14.06 (6.28)	27	14.78 (6.97)	22	15.77 (7.67)
	Control	35	12.06 (4.81)	27	11.56 (5.91)	22	11.14 (6.20)		
** Stigma **									
	Web only	38	10.26 (5.36)	27	7.37 (5.10)	22	8.09 (5.76)	21	6.57 (4.13)
	Web with tracking	43	11.16 (4.63)	20	10.80 (6.43)	20	9.60 (5.83)	14	10.57 (7.65)
	Tracking only	36	12.61 (6.07)	31	12.42 (5.45)	26	11.58 (5.01)	22	12.09 (5.75)
	Control	34	11.62 (5.61)	27	11.96 (4.68)	22	13.73 (5.39)		
** Depression literacy **									
	Web only	37	4.27 (2.46)	26	5.96 (2.18)	21	5.57 (2.09)	19	6.47 (2.37)
	Web with tracking	44	4.09 (2.06)	20	5.35 (2.35)	19	5.53 (2.50)	13	5.46 (2.76)
	Tracking only	36	4.67 (2.19)	31	4.42 (2.09)	24	4.33 (2.06)	18	5.00 (1.61)
	Control	34	4.88 (2.33)	27	5.19 (2.80)	20	4.95 (2.67)		
** CBT literacy **									
	Web only	37	4.35 (1.89)	27	5.67 (1.62)	22	5.77 (1.27)	21	6.33 (1.43)
	Web with tracking	42	4.26 (2.08)	20	5.80 (1.40)	20	5.80 (2.02)	14	5.79 (1.76)
	Tracking only	35	5.06 (1.81)	31	4.10 (1.80)	25	4.48 (1.71)	21	4.67 (1.98)
	Control	34	4.29 (1.80)	27	4.41 (1.95)	20	4.35 (1.98)		

^a ^Different numbers are due to incomplete responses and unequal dropout between conditions.

### Correlations Between Secondary Outcomes and Depression Symptoms at Preintervention, Postintervention, and Follow-ups

A strong negative correlation was found between preintervention depression symptoms and quality of life (*r *= –.52, *P *< .001). We found no other significant correlations: hazardous alcohol use (*r *= .04, *P *= .68), stigma (*r *= .15, *P *= .06), depression literacy (*r *= .15, *P *= .07), and CBT literacy (*r *= –.04, *P *= .63).

 At postintervention, depression symptoms and quality of life were negatively correlated (*r *= –.65, *P *< .001), but no other significant correlations were found for hazardous alcohol use (*r *= .09, *P *= .38), stigma (*r *= .15, *P *= .13), depression literacy (*r *= .08, *P *= .44), and CBT literacy (*r *= –.15, *P *= .12).

 The pattern of results was similar at the 6- and 12-month follow-ups. Quality of life was negatively correlated with depression at 6 months (*r *= –.77, *P *< .001) and 12 months (*r *= –.69, *P *< .001). At 12 months, stigma was positively associated with depression symptoms (*r *= .29, *P *= .03). No other significant correlations were found: hazardous alcohol use (6 months: *r *= .06, *P *= .58; 12 months: *r *= .15, *P *= .28), stigma (6 months: *r *= .17, *P *= .11), depression literacy (6 months: *r *= .06, *P *= .60; 12 months: *r *= –.07, *P *= .63), and CBT literacy (6 months: *r *= –.18, *P *= .10; 12 months: *r *= –.17, *P *= .20).

### Hazardous Alcohol Use

At preintervention, postintervention, 6-month follow-up, and 12-month follow-up, 49/153 (32%), 22/99 (22%), 17/88 (19%), and 14/57 (25%) participants reported a hazardous level of alcohol use, respectively. There were no significant differences in the proportions of participants reporting hazardous use between conditions at any time point. Table 1 shows mean AUDIT scores for each intervention on each occasion. Planned comparisons of parameters in the random intercept Poisson regression model showed that postintervention participants who received the Web-only or the Web with tracking interventions had a greater decline than those in the tracking-only (χ^2^_1 _= 4.7, *P *= .03; χ^2^_1 _= 10.4, *P *< .01) or control condition (χ^2^_1 _= 5.8, *P *= .02; χ^2^_1 _= 11.3, *P *< .01). Differences between the two Web interventions and between the tracking-only and the control condition were not significant (χ^2^_1 _= 0.5, *P *= .50; χ^2^_1 _= 0.3, *P *= .61). At the 6-month follow-up, change from preintervention was significantly different for the Web-only and Web with tracking conditions compared with the control group (χ^2^_1 _= 4.0, *P *< .05; χ^2^_1 _= 5.4, *P *= .02). There were no significant differences between conditions at the 12-month follow-up.

At postintervention, effect sizes were 0.23 (95% CI –0.32 to 0.79) for the Web-only condition and 0.26 (95% CI –0.39 to 0.91) for the Web with tracking condition, compared with the control condition. Compared with tracking only, effect sizes were 0.27 (95% CI –0.25 to 0.78) for the Web-only condition and 0.31 (95% CI –0.31 to 0.93) for the Web with tracking condition.

### Quality of Life

The occasion-by-condition interaction was also significant for quality of life (*F*_8,96.4 _= 2.39, *P *= .02). At postintervention, participants who received the Web with tracking and Web-only interventions has significantly greater improvements in quality of life than participants in the control condition, but not the tracking-only condition (Table 2). At 6 months, the Web with tracking (contrast estimate = 5.12, 95% CI 1.81–8.45, *P *= .003) and Web-only (contrast estimate = 5.06, 95% CI 1.93–8.18, *P *= .002) conditions remained superior to the control condition. No significant differences were observed between conditions at 12 months.

At postintervention, effect sizes were 0.81 (95% CI 0.25–1.36) for the Web-only condition and 0.63 (95% CI –0.02 to 1.27) for the Web with tracking condition, compared with the control condition. Compared with tracking only, effect sizes were 0.41 (95% CI –0.11 to 0.92) for the Web-only condition and 0.22 (95% CI –0.39 to 0.83) for the Web with tracking condition.

**Table 2:** Contrast estimates, 95% confidence intervals (CI), test of significance values, and effect size estimates for contrasts of pre- to postintervention change between intervention and control conditions.

Outcome	Statistic				
		Web only vs control	Web only vs tracking only	Web with tracking vs control	Web with tracking vs tracking only
Quality of life	Contrast estimate	4.20	2.31	3.88	1.98
	95% CI	1.70–6.70	–0.08 to 4.70	0.99–6.76	–0.81 to 4.78
	*P *value	.001	.06	.009	.16
Stigma	Contrast estimate	–2.19	–.97	–.22	.10
	95% CI	–4.35 to –0.03	–3.05 to 1.11	–2.59 to 2.14	–1.30 to 3.30
	*P *value	.047	.36	.85	.39
Depression literacy	Contrast estimate	0.61	1.07	1.16	1.61
	95% CI	–0.42 to 1.65	0.07–2.07	0.03–2.29	0.52–2.71
	*P *value	.24	.04	.045	.004
CBT literacy	Contrast estimate	1.10	1.87	1.79	2.56
	95% CI	0.31–1.88	1.10–2.63	0.94–2.64	1.73–3.39
	*P *value	.01	<.001	<.001	<.001

### Stigma

The interaction of condition and occasion was nonsignificant for stigma (*F*_8,96.5 _= 1.73, *P *= .10). Nonetheless, we found several between-condition contrasts to be significant. At postintervention, participants in the Web-only condition showed significantly lower levels of stigma than participants in the control condition. At 6 months, stigma was significantly reduced in participants in the Web-only condition (contrast estimate = –3.29, 95% CI –5.97 to –0.61, *P *= .02) and the Web with tracking condition (contrast estimate = –2.88, 95% CI –5.71 to –0.05, *P *= .046), relative to participants in the control condition. No significant differences were found between conditions at the 12-month follow-up.

At postintervention, effect sizes were 0.94 (95% CI 0.38–1.50) for the Web-only condition and 0.17 (95% CI –0.42 to 0.77) for the Web with tracking condition, compared with the control condition. Compared with tracking only, effect sizes were 0.96 (95% CI 0.41–1.50) for the Web-only condition and 0.24 (95% CI –0.34 to 0.82) for the Web with tracking condition.

### Depression Literacy

The interaction of condition and occasion was also nonsignificant for depression literacy (*F*_8,96.5 _= 1.75, *P *= .10). However, at postintervention, contrasts revealed that participants who received the Web with tracking intervention had significantly higher levels of depression literacy than participants in the control and tracking-only conditions. Participants in the Web-only condition also had significantly improved depression literacy compared with participants in the tracking-only condition. At 6 months, depression literacy remained higher in participants in the Web with tracking condition than in participants in the control condition (contrast estimate = 1.64, 95% CI 0.30–3.00, *P *= .02). No significant differences were found between conditions at 12 months.

At postintervention, effect sizes were 0.31 (95% CI –0.23 to 0.85) for the Web-only condition and 0.01 (95% CI –0.58 to 0.61) for the Web with tracking condition, compared with the control condition. Compared with tracking only, effect sizes were 0.73 (95% CI 0.19–1.26) for the Web-only condition and 0.37 (95% CI –0.21 to 0.96) for the Web with tracking condition.

### CBT Literacy

We found a significant condition-by-occasion interaction for CBT literacy (*F*_8,101.9 _= 6.29, *P *< .001). At postintervention, participants who received both Web interventions had significantly higher CBT literacy scores than participants in the control and tracking-only conditions. At 6 months, these improvements were maintained such that CBT literacy was higher in participants in the Web-only (contrast estimate = 1.24, 95% CI 0.33–2.15, *P *= .008) and Web with tracking (contrast estimate = 1.71, 95% CI 0.76–2.67, *P *= .001) conditions relative to the control condition. CBT literacy was also higher in the Web-only (contrast estimate = 1.48, 95% CI 0.61–2.37, *P *= .001) and Web with tracking (contrast estimate = 1.96, 95% CI 1.03–2.88, *P *< .001) conditions relative to the tracking-only condition. At 12 months, participants in both the Web-only (contrast estimate =1.93, 95% CI 0.94–2.92, *P *< .001) and Web with tracking (contrast estimate = 1.91, 95% CI 0.82–3.00, *P *= .001) conditions showed higher CBT literacy than participants in the tracking-only condition.

At postintervention, effect sizes were 0.71 (95% CI 0.16–1.26) for the Web-only condition and 0.80 (95% CI 0.18–1.42) for the Web with tracking condition, compared with the control condition. Compared with tracking only, effect sizes were 0.92 (95% CI 0.37–1.46) for the Web-only condition and 1.03 (95% CI 0.41–1.64) for the Web with tracking condition.

## Discussion

Analysis of the secondary outcomes of this trial suggests that Web-based CBT is effective in the short-term minimization of hazardous alcohol use, and in the immediate- and longer-term improvement of quality of life and CBT literacy. For these outcomes, weekly telephone tracking provided by a lay telephone counselor did not confer any advantage over delivery of the Web intervention alone. This result is consistent with that obtained in the analysis of the primary outcome of depression for this trial [7], where we found that Web-based CBT without telephone tracking was as effective as the intervention with telephone tracking. Primary outcome analyses also revealed that telephone tracking was associated with significantly greater dropout in the Web with tracking condition than in other conditions. As might be expected, given that the same participants were involved, dropout was higher in the Web with tracking condition on the secondary outcome measures as well.

At preintervention, average levels of alcohol use approached the cut-off indicating hazardous use. Participants who received the Web intervention significantly reduced their alcohol use, suggesting that Web-based CBT for depression may be effective in the prevention of hazardous alcohol use. CBT-based interventions have been shown to be effective in the treatment of alcohol use disorders. However, little is known about the mechanisms of action that underpin how and why CBT is effective. It has been hypothesized that CBT promotes the acquisition of cognitive and behavioral coping skills that enable individuals to manage the life stress and alcohol cues that maintain excessive drinking [36]. However, a review by Morgenstern and Longabaugh did not find evidence to support the mediating role of coping skills [36]. More recent investigations of computer-based CBT for substance use disorders have revisited this hypothesis, suggesting that it may be the quality, not quantity, of coping skills acquired through CBT that leads to reductions in hazardous alcohol use [37].

 The Web intervention was also effective in improving quality of life. We found a strong negative correlation between depression and quality of life at preintervention, and improvement in quality of life may be partly associated with the observed reductions in depression symptoms. However, quality of life reflects the broader construct of well-being that includes variables such as satisfaction with general health, finances, social relationships, and activities of daily living. It is possible that the alleviation of depression symptoms, or the acquisition of cognitive and behavioral strategies, or both, are associated with an enhanced ability to effect change in dissatisfying life circumstances or to adopt a more helpful or realistic view of existing circumstances. In any case, the findings show that the effects of the intervention were broader than those of depression symptoms.

 The intervention was not effective overall in improving depression literacy or in reducing stigma, although some contrast effects were found. Interventions that focus on providing information about mental illness have been shown to improve both depression literacy and stigma [19]. The length of the psychoeducational component of the current intervention (1 week) was probably not sufficient to produce meaningful change in knowledge of depression, a factor that may also account for the absence of differential change in depression stigma. A trial involving a larger “dose” of Web-based psychoeducation than the current trial (5 weeks) obtained both significant increases in depression literacy and significant reductions in stigma at postintervention [21]. An additional explanation may be the use of the shortened version of the depression literacy scale in the current trial. The hypothesis that stigma reduction is mediated by an improvement in depression literacy has been examined previously [21]. However, in the current study, the intervention was not found to be effective for either depression literacy or stigma, violating the conditions required to investigate mediation using the causal-steps approach proposed by Baron and Kenny [38].

The current intervention was effective, however, in improving knowledge of the key principles of CBT, which is expected, given that this was the predominant focus of the intervention. The sustained improvement in CBT knowledge observed at the 6- and 12-month follow-ups suggests that participants engaged with the intervention at a depth that created lasting memory for the therapeutic concepts contained in the program.

### Limitations

We acknowledged that greater dropout in the Web with tracking condition may have inflated the effects observed in this condition. It is noteworthy, however, that dropout was not greater in the Web-only condition, so that the finding of differential dropout from the Web with tracking condition does not undermine the findings suggesting that the Web intervention conditions are more effective than either the tracking-only or control conditions.

It is possible that differences in the amount of Lifeline use between conditions throughout the trial may have influenced the findings, particularly if participants in the intervention conditions used the service more. However, those in the active treatment conditions (Web only and Web with tracking) declined to use the service during the intervention period, and were significantly less likely than those in the control condition to use the Lifeline service frequently (calling 3 or more times in a 1-month period) at postintervention.

Further, as noted in our report of the primary outcomes of this trial [7], uptake of the trial and intervention completion rates were lower in our study than in some other trials of Web-based treatments for depression [39]. The reasons for this are unclear. Users of telephone counseling services may experience more stressful events that preclude trial completion or may prefer to manage their own health. The current trial was a true effectiveness trial employing the telecounseling organization’s existing volunteer workforce for recruitment and tracking, and hence would be expected to be associated with greater recruitment and adherence issues relative to the more controlled environment of an efficacy study [40].

The current study found evidence of efficacy for the secondary outcomes examined, but we did not investigate the mediating variables through which the intervention was effective. Pre–posttest designs do not fully allow for examination of mediation or causal chains. Studies including more frequent observations are needed to examine such effects, and studies of this type need not be controlled trials. Moreover, in some cases, the relationships between key variables of interest were nonsignificant, and thus we could not conduct mediator analyses.

### Conclusion

This is the first study to demonstrate that a Web-based psychoeducation and CBT intervention introduced through a telephone helpline is effective in minimizing hazardous alcohol use, and in improving quality of life and knowledge of CBT. The results highlight the potential for low-cost, low-intensity Web-based interventions to produce change in outcomes beyond those that the intervention is primarily designed to produce.

## Abbreviations

AUDIT: Alcohol Use Disorders Identification Test
CBT: cognitive behavior therapy
WHOQOL: World Health Organization Quality of Life
